# Factors associated with vitamin D deficiency in health care workers exposed to SARS-CoV-2: a cross-sectional study

**DOI:** 10.3389/fnut.2024.1440185

**Published:** 2024-07-24

**Authors:** Miguel Angel Villasis-Keever, Jessie Nallely Zurita-Cruz, Juan Garduño-Espinosa, Mardya López-Alarcón, Aly Sugey Barradas Vázquez, María Guadalupe Miranda-Novales, Israel Parra-Ortega, Briseida López-Martinez, Heladia García, Miguel Klünder-Klünder

**Affiliations:** ^1^Analysis and Synthesis of the Evidence Research Unit, National Medical Center XXI Century, Instituto Mexicano del Seguro Social, Mexico City, Mexico; ^2^Facultad de Medicina Universidad Nacional Autónoma de México, Hospital Infantil de México Federico Gómez, Mexico City, Mexico; ^3^Evidence-Based Medicine Unit, Hospital Infantil de México Federico Gómez, Ministry of Health (SSA), Mexico City, Mexico; ^4^Clinical Research Division of the Health Research Coordination, National Medical Center XXI Century, Instituto Mexicano del Seguro Social, Mexico City, Mexico; ^5^Auxiliary Diagnostic Services, Hospital Infantil de México Federico Gómez, Ministry of Health (SSA), Mexico City, Mexico; ^6^Epidemiological Research Unit in Endocrinology and Nutrition, Hospital Infantil de México Federico Gómez, Ministry of Health (SSA), Mexico City, Mexico City, Mexico

**Keywords:** 25-hydroxy vitamin D, vitamin D deficiency, health care worker, obesity, type 2 diabetes

## Abstract

**Introduction:**

Globally, up to 76.6% of the population may be affected by vitamin D (VD) deficiency, which has been linked to increased morbidity and mortality from COVID-19. This underscores the importance of further research into VD supplementation, particularly for health care workers, who are at higher risk due to indoor work environments and dietary challenges associated with shift schedules.

**Objective:**

This study aimed to identify factors associated with VD deficiency in Mexican health care workers exposed to SARS-CoV-2.

**Materials and methods:**

We conducted a cross-sectional study from June 2020 to January 2021 among frontline health care workers treating hospitalized COVID-19 patients. Blood samples were collected to measure 25-hydroxy VD levels via radioimmunoassay. We also assessed previous COVID-19 infection and comorbidities that could influence VD levels.

**Results:**

The study included 468 health care workers. The median serum VD concentration was 16.6 ng/mL. VD deficiency was found in 69.4% (*n* = 325) of participants, while only 5.1% (*n* = 24) had normal levels. Those with type 2 diabetes (13.3 ng/mL vs. 17.1 ng/mL) or obesity (15.7 ng/mL vs. 17.1 ng/mL) had significantly lower VD levels than their counterparts (*p* < 0.001 and *p* = 0.049, respectively). No significant differences were found among participants with high blood pressure. Multivariate analysis revealed that type 2 diabetes was independently associated with VD deficiency.

**Conclusion:**

There is a high prevalence of VD deficiency among health care workers, which is potentially linked to both personal health factors and occupational conditions.

## Introduction

Vitamin D (VD) deficiency poses a significant global health challenge, with research indicating a prevalence rate of up to 76.6%, underscoring substantial regional variability and the impact of diverse determinant factors (1). Certain occupational groups exhibit a heightened prevalence of VD deficiency. This issue is partly linked to limited sun exposure inherent to specific job roles, particularly those conducted predominantly indoors or within shift work schedules, including night shifts (2). Additionally, those working night or rotating shifts are prone to less healthy dietary patterns. This tendency is influenced by a lack of suitable nutritional options and restricted eating times due to long working hours, leading to diets high in ultra-processed foods, meats, fats, and alcoholic beverages (3). Such dietary habits, combined with the demands of shift work, may contribute to the development of digestive disorders, sleep disturbances, and comorbidities (4). These risk factors, including limited sun exposure, inadequate diet, and obesity, which are inherent to the health care profession, significantly contribute to VD deficiency (5, 6).

During the COVID-19 pandemic, populations in areas with a high prevalence of VD deficiency experienced increased morbidity and mortality related to COVID-19 infection. This finding supports further studies on the prevalence of VD deficiency and the benefits of VD supplementation across various pathologies, from cardiometabolic disorders to viral infections (7). During the pandemic, the working conditions of health care workers worsened, likely due to the increase in indoor activities—both work-related and personal—heightening the risk of VD deficiency (8).

VD affects various body systems beyond bone health, including the immune system, where it modulates immune responses; the cardiovascular system, which influences heart and blood vessel functions; the endocrine system, particularly insulin secretion and glucose metabolism; and the nervous system, which affects brain function and mental health. These roles highlight the importance of VD in overall health and disease prevention (9).

Therefore, this study aimed to identify factors associated with VD deficiency in Mexican health care workers exposed to SARS-CoV-2. This information may strengthen the importance of vitamin D supplementation in this high-risk group.

## Materials and methods

This cross-sectional study was carried out between June 2020 and January 2021 at four tertiary care hospitals treating COVID-19 patients in Mexico City. Participants were enrolled during the period from June 15 to December 15, 2020, encompassing the summer and fall seasons. The inclusion criteria were frontline health care workers who were hospitalized with COVID-19, who underwent confirmatory testing for SARS-CoV-2 due to suspected symptoms or due to having unprotected contact with a suspected case, and who had a blood test for 25-hydroxy vitamin D (25[OH]D) levels. The exclusion criteria included subjects who were already receiving VD or other vitamin supplements and those whose 25[OH]D samples were not processed.

Data from health care workers without COVID-19 were obtained from a previous clinical trial registered at ClinicalTrials.gov (#NCT04535791), and these data were used to analyze the efficacy and safety of VD supplementation in preventing SARS-CoV-2 infection (10).

Participants were divided into two groups: those with VD deficiency (25[OH]D levels <20 ng/mL) and those without (25[OH]D levels ≥20 ng/mL) (11).

As previously described (10), all participants underwent simultaneous serum sampling for 25[OH]D determination and RT–PCR COVID-19 testing, which occurred as part of the RCT selection process. Anthropometric measurements and blood pressure assessments were performed on the participants. Obesity was defined as a body mass index (BMI) >30. Previous diagnoses of type 2 diabetes (T2D) and high blood pressure (HBP) were recorded as comorbidities. HBP was defined as a persistent elevation of blood pressure above systolic pressure ≥ 140 mmHg or diastolic pressure ≥ 90 mmHg (12), and T2D was defined as fasting plasma glucose levels >126 mg/dL or 2-h values in the oral glucose tolerance test of >200 mg/dL (13). A subject was classified as having a positive SARS-CoV-2 infection if they presented a positive real-time polymerase chain reaction (RT–PCR) test.

The protocol was approved by the institutional review boards of the Instituto Mexicano del Seguro Social (CNIS # R-2020-785-090) and Hospital Infantil de Mexico Federico Gómez (HIM-2020-045). All participants provided written informed consent after the procedures were explained according to the protocol.

### Laboratory procedures

#### Vitamin D determination

The 25(OH)D concentrations were determined at the Hospital Infantil de Mexico Clinical Laboratory using the Abbot brand chemiluminescence technique with Architect 1,000 equipment. According to the serum levels, a VD deficiency was defined as <20 ng/mL, VD insufficiency was defined as 20–29.99 ng/mL, and a normal level was defined as >30 ng/mL (11, 14).

### Statistical analysis

Using the Kolmogorov–Smirnov test, it was determined that the quantitative variables did not have a Gaussian distribution, so they are presented as median with their respective 95% confidence intervals (95% CIs). Qualitative variables are expressed as proportions and frequencies.

Patients were categorized into two groups: those with and without VD deficiency. VD levels were compared across participants based on their comorbidities and type of personnel. Additionally, serum VD concentrations were compared among factors that could influence levels, such as obesity, T2D, HBP, age, and sex.

To assess differences between groups, we applied the Mann–Whitney U test or Kruskal-Wallis for quantitative variables and the chi–square test for qualitative variables.

Multiple logistic regression analysis was conducted to determine the associations of VD levels with age, male sex, HBP, T2D, and obesity. All the statistical analyses were performed using STATA version 12.0.

## Results

Among the 590 potential candidates for inclusion, 122 were excluded: 72 did not meet the inclusion criteria, 18 had samples that were not processed for serum vitamin D levels, and 32 were health care workers who declined to participate. Among the 468 health care workers included, the median age was 40 years, and there was a predominance of female participants (65.4%). Nursing was the most common type of personnel (35.5%), followed by doctors (20.5%) and laboratory personnel (17.7%).

As shown in Table 1, 197 (42.1%) participants had one or more comorbidities, and HBP was the most common (*n* = 145, 31.0%). In total, 151 patients were positive for SARS-CoV-2 (32.3%).

**Table 1:** General characteristics of the health-care workers included.

Participants, No. (%)				
		Vitamin D classification		
Characteristic	Total *n* = 468	<20 ng/mL *n* = 325 (69.4%)	> 20 ng/mL *n* = 143 (30.6%)	*p*-value
Sex				
Female	306 (65.4)	208 (64.0)	98 (68.5)	0.343
Male	162 (34.6)	117 (36.0)	45 (31.5)	
Age, y				
Median (CI 95%)	40 (40–42)	41 (40–43)	39 (38–42)	0.377
Risk factors				
High blood pressure	145 (31.0)	99 (30.5)	46 (32.2)	0.713
Type 2 Diabetes	49 (16.3)	43 (20.8)	6 (6.4)	**0.001**
Obesity	126 (26.9)	96 (29.5)	30 (21.0)	0.054
Comorbidities (≥ 1 of the above)	197 (42.1)	147 (45.2)	50 (35.0)	**0.038**
25 hydroxy- Vitamin D, ng/ml				
Median (CI 95%)	16.6 (17.0–18.2)	14.4 (13.9–14.6)	23.6 (24.3–26.4)	**<0.001**
<20	325 (69.4)	325 (100)	-	**0.001**
20–29.9	119 (25.5)	-	119 (83.2)	
≥ 30	24 (5.1)	-	24 (16.8)	
Type of personnel				0.085
Nurses	166 (35.5)	120 (36.9)	46 (32.2)	
Doctors	96 (20.5)	60 (18.5)	36 (25.2)	
Laboratory workers	83 (17.7)	51 (15.7)	32 (22.4)	
Others	83 (17.7)	63 (19.4)	20 (14.0)	
Orderlies and cleaning staff	40 (8.6)	31 (9.5)	9 (6.3)	
Infection due to SARS-CoV-2				
Positive	151 (32.3)	117 (36.0)	34 (23.8)	**0.009**

BMI, body mass index; CI 95%, confidence interval 95%. BMI is calculated as person’s weight in kilograms divided by the square of height in meters. Bold values means statistical significance.

**Figure 1:**
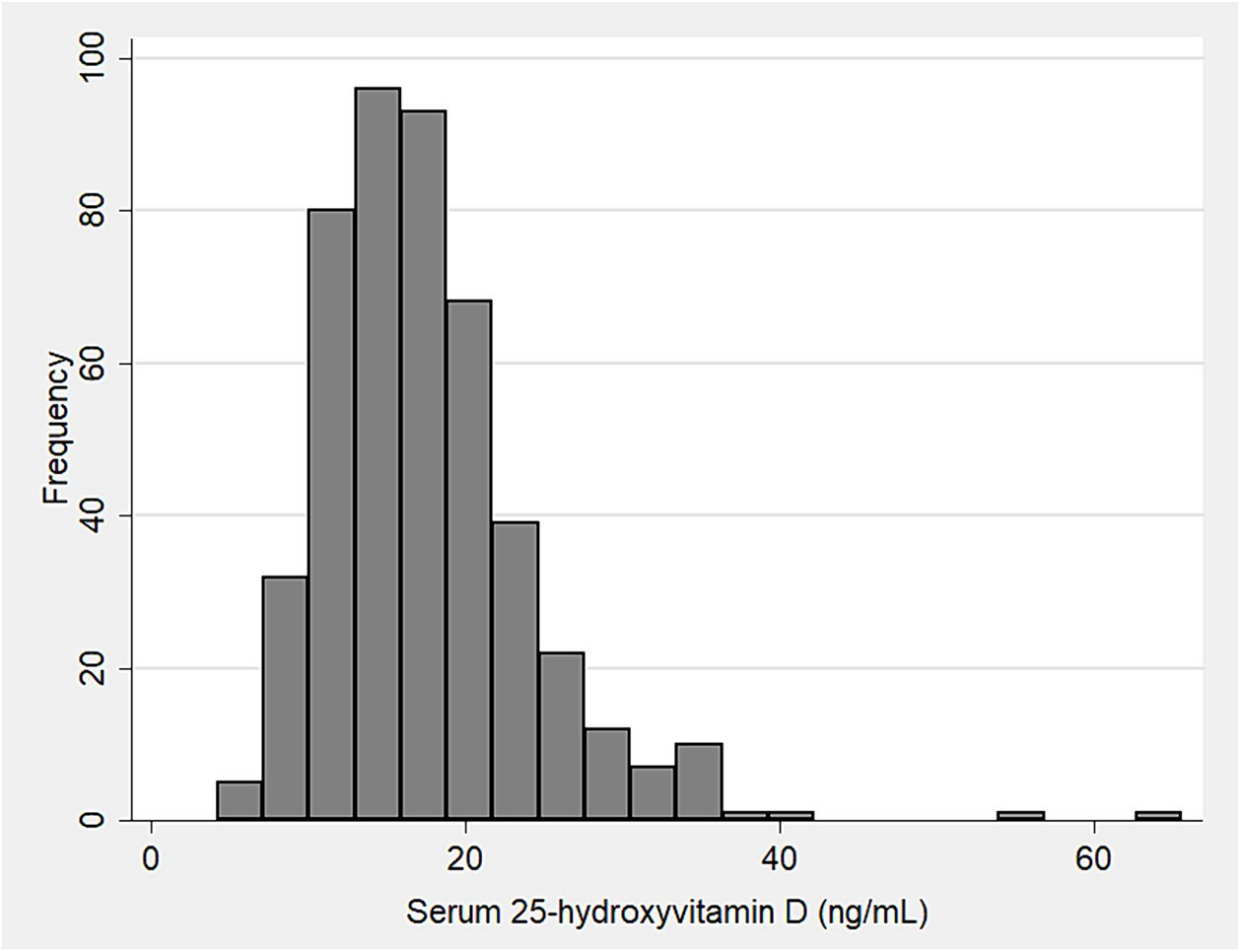
Histogram showing the distribution of serum 25-hydroxy vitamin D levels in 468 health-care workers.

The distribution of VD levels is shown in Figure 1; the median VD level among all health care workers was 16.6 ng/mL (95% CI 17.0–18.2 ng/mL). Among the 24 health care workers with sufficient VD levels, the median age was 39 years (95% CI: 38–46). Of these, 4.2% (*n* = 1) had DM2, 37.5% (*n* = 9) had hypertension, and 20.8% (*n* = 5) were obese.

**Figure 2:**
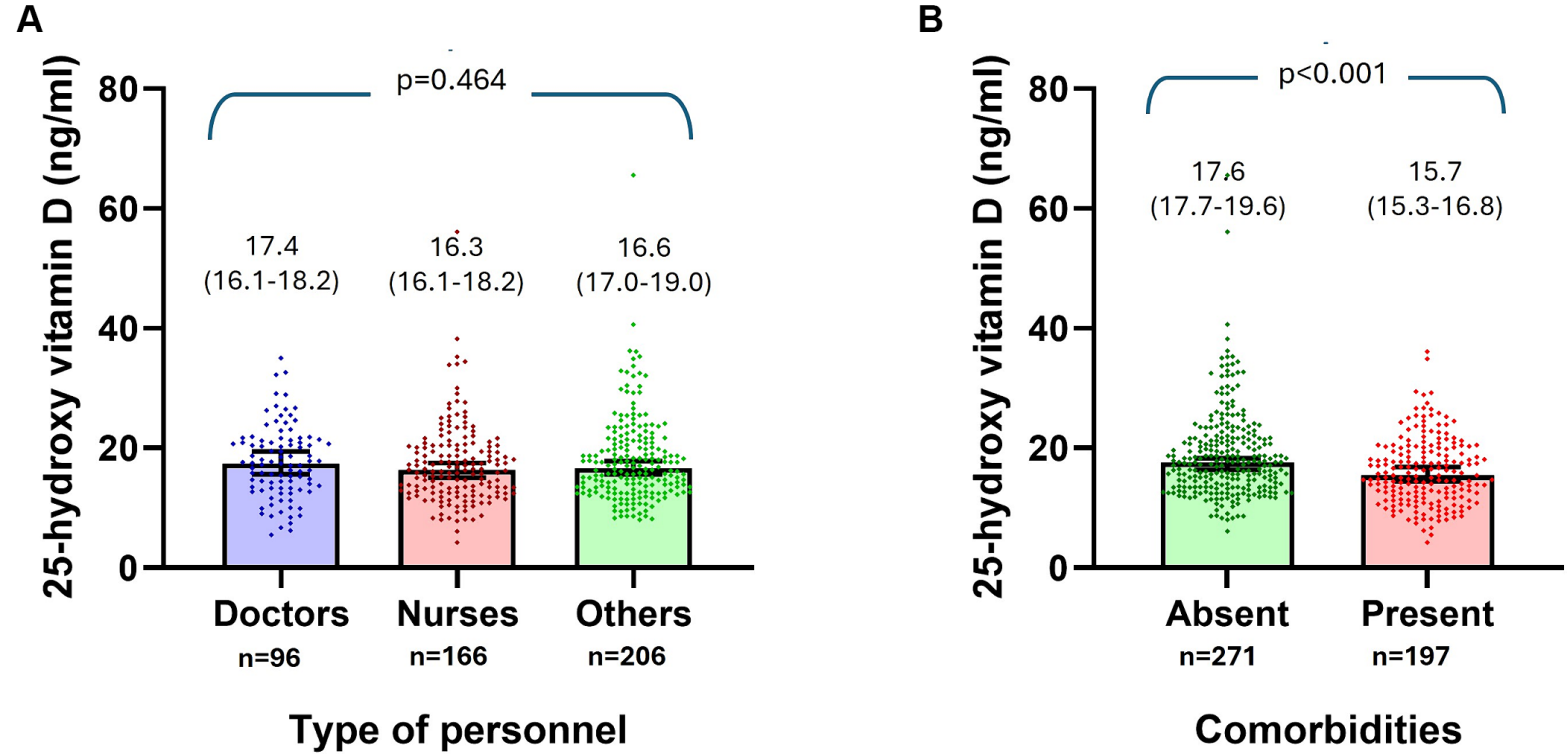
Comparison of serum levels of 25-hydroxy vitamin D (ng/ml) according to the type of personnel **(A)**, and by the presence or not of comorbidities **(B)**.

There was no difference in VD levels according to age or sex, nor by type of health personnel (Figure 2A). However, when comparing between participants with and without VD deficiency (Table 1), a smaller proportion of doctors (18.5% vs. 25.5%) and laboratory workers (15.7% vs. 22.4%) had VD deficiency compared to nurses (36.9% vs. 32.2%), but this difference was not statistically significant. By contrast, participants with T2D and other comorbidities had a statistically higher frequency of VD deficiency (*p* < 0.05), as shown in Table 1.

Serum VD concentrations were also compared among patients according to factors that could influence serum levels. Subjects with T2D (13.3 ng/mL vs. 17.1 ng/mL, *p* < 0.001), obesity (15.7 ng/mL vs. 17.1 ng/mL, *p* = 0.047), and comorbidities (15.7 ng/mL vs. 17.6 ng/mL, *p* < 0.001) had lower VD levels than those without these factors (Figure 2B). No significant differences were found among participants with HBP (Table 2).

**Table 2:** Comparison of serum levels of 25-hydroxy vitamin D (ng/ml) according to the characteristics of healthcare workers.

	Comorbidities		
	Present	Absent	
Comorbidities	Median (95% confidence interval)		*p*-value
Male sex (*n* = 162)	15.8 (16.4–18.5)	17.0 (16.9–18.4)	0.349
Obesity (*n* = 126)	15.7 (15.5–17.7)	17.1 (17.2–18.7)	**0.047**
Type 2 diabetes (*n* = 57)	13.3 (12.8–15.6)	17.1 (17.4–18.7)	**<0.001**
High blood pressure (*n* = 145)	16.9 (16.5–19.0)	16.5 (16.9–18.2)	0.786
Comorbidities (*n* = 197)	15.7 (15.3–16.8)	17.6 (17.9–19.6)	**<0.001**

Bold values means statistical significance.

According to the multivariate analysis, T2D (OR 3.40, CI 95%: 1.43 to 8.05, *p* = 0.005) was associated with VD deficiency, adjusted for age, sex, the presence of obesity, and HBP (Table 3).

**Table 3:** Logistic regression analysis to identify factors related to deficiency vitamin D.

	Adjusted OR	95% confidence interval	*p*-value
Age (years)	0.99	0.97 to 1.01	0.924
Male sex	1.18	0.77 to 1.81	0.438
Type 2 Diabetes	3.40	1.43 to 8.05	**0.005**
Obesity	1.44	0.88 to 2.34	0.141
High blood pressure	0.08	0.52 to 1.25	0.346

Bold values means statistical significance.

## Discussion

The high prevalence of VD deficiency among Mexican health care workers exposed to SARS-CoV-2 observed in our study is alarming, with 69.4% showing low serum levels. This rate is significantly higher than that seen in the general population but aligns with findings from studies on high-risk occupational groups and individuals with conditions such as obesity and diabetes (2, 15). This rate of VD deficiency, which is higher than the global average of 50%, underscores the severity of the issue. In the Americas, the prevalence of VD deficiency has reached 77% (16). Specifically, in Mexico, the National Health and Nutrition Survey 2006 (ENSANUT) reported a VD deficiency and insufficiency rate of 30% in adults, with Mexico City (area where this study was developed) showing a higher rate than other regions, at 43.1% (17). During the first year of the COVID-19 pandemic, health care workers faced long hours (which reduced sun exposure) and a shortage of medications (including cholecalciferol) and consumed low levels of VD-rich foods, leading to increased rates of VD deficiency. It is crucial to maintain optimal VD concentrations, which, according to the Society of Endocrinology, should be above 30 ng/mL (14, 18). Our findings are similar to those recently reported by Ito et al.; they conducted a study to examine the prevalence of VD deficiency among healthcare workers following the onset of the COVID-19 pandemic (19). They identified that 9.2% of the subjects had adequate VD levels, whereas our data revealed that only 5.1% had adequate VD levels (>30 ng/mL).

Studies focusing on VD deficiency across different occupational and health risk groups have documented varying prevalence rates. For instance, shift workers and indoor workers experience similar trends of VD deficiency due to comparable environmental and work conditions that limit sun exposure, with deficiency rates between 78 and 80% compared to 48% for outdoor workers (2, 20). Recent studies have reported that the prevalence of VD deficiency among health care workers ranges from 45 to 51% (19, 21). These trends, observed across occupations with limited outdoor activity, highlight the occupational risk associated with VD deficiency. These findings emphasize the critical role of occupational factors in VD levels, suggesting that regular screening for VD levels in high-risk occupational groups should be considered in clinical practice guidelines and public health initiatives to prevent adverse health outcomes linked to VD deficiency.

Health care workers often endure prolonged periods indoors with limited exposure to natural sunlight, which is the primary source necessary for synthesizing VD in the skin. This issue is compounded in roles that involve night shifts or extensive indoor duties. The deficiency in VD is due to low UVB exposure and a secondary decrease in vitamin synthesis in the skin with aging (22, 23), as the precursor to VD, 7-dehydrocholesterol, decreases by approximately 50% between the ages of 20 and 80 (23).

Obesity and diabetes merit particular attention among the factors that influence VD levels. The literature presents contradictory results regarding the impact of obesity on VD deficiency (24). However, VD deficiency becomes more apparent in obese patients when diabetes mellitus is also present. A study conducted by Atia et al. (25) revealed that the prevalence of VD deficiency was significantly greater in individuals with prediabetes than in those without prediabetes (38.5% vs. 25.5%). In obese individuals, VD tends to be sequestered within adipose tissue, reducing its availability in the bloodstream. This biological trapping of VD in fat cells diminishes its overall active circulation (26). In the context of diabetes, the disease’s impact on renal function and liver enzymes can interfere with the hydroxylation processes that are essential for the vitamin’s activation. High glucose levels, which are common in diabetes, may further impair these processes, complicating the metabolic pathway required for converting VD to its active form (27, 28). These interconnected mechanisms highlight a compounded risk where occupational and personal health factors converge, leading to increased vulnerability to VD deficiency.

Study limitations include the inability to specify the work schedules of the included workers, as shifts varied during the pandemic. The study did not analyze the seasons during which participants were included; however, only non-winter seasons, namely, summer and autumn, were considered; winter, which is associated with decreased serum VD levels, was not included. The major limitation was the cross-sectional study design, which could not establish a causal relationship between VD deficiency and modifying factors such as obesity and diabetes.

Given the high prevalence of VD deficiency among health care workers, particularly those with prolonged indoor exposure and minimal sunlight, it is imperative to routinely evaluate serum VD levels. For health care workers, especially those positive for SARS-CoV-2, the benefits of VD supplementation could extend beyond correcting the deficiency—it might also enhance immune response capabilities against viral infections. Considering the broader implications, maintaining adequate vitamin D levels is crucial for infectious disease prevention and general health maintenance within health care settings.

In conclusion, the frequency of low VD values is high among health care workers, and TD2 was a significant risk factor associated with these values. This study underscores the urgent need for systematic VD deficiency screening and tailored VD supplementation strategies. Such measures are crucial not only for the health of individual workers but also for supporting the operational integrity and resilience of health care systems facing ongoing public health challenges.

## Data availability statement

The original contributions presented in the study are included in the article/supplementary material, further inquiries can be directed to the corresponding author.

## Ethics statement

The studies involving humans were approved by The protocol was approved by the institutional review boards of the Instituto Mexicano del Seguro Social (CNIS # R-2020-785-090) and Hospital Infantil de Mexico Federico Gómez (HIM-2020-045). The studies were conducted in accordance with the local legislation and institutional requirements. The participants provided their written informed consent to participate in this study.

## Author contributions

MV-K: Conceptualization, Funding acquisition, Supervision, Validation, Writing – review & editing. JZ-C: Data curation, Formal analysis, Investigation, Writing – original draft, Writing – review & editing. JG-E: Funding acquisition, Supervision, Writing – review & editing. ML-A: Resources, Supervision, Writing – review & editing. AB: Data curation, Investigation, Writing – review & editing. MM-N: Formal analysis, Investigation, Writing – review & editing. IP-O: Formal analysis, Investigation, Writing – review & editing. BL-M: Investigation, Writing – review & editing. HG: Writing – review & editing, Investigation. MK-K: Funding acquisition, Supervision, Writing – review & editing.
